# Distinct Responses of the Nitrogen-Fixing Marine Cyanobacterium *Trichodesmium* to a Thermally Variable Environment as a Function of Phosphorus Availability

**DOI:** 10.3389/fmicb.2019.01282

**Published:** 2019-06-11

**Authors:** Pingping Qu, Fei-Xue Fu, Joshua D. Kling, Megan Huh, Xinwei Wang, David A. Hutchins

**Affiliations:** ^1^Department of Biological Sciences, University of Southern California, Los Angeles, CA, United States; ^2^Department of Preventive Medicine, Keck School of Medicine, University of Southern California, Los Angeles, CA, United States; ^3^School of Life Sciences, Xiamen University, Xiamen, China

**Keywords:** ocean warming, thermal variation, *Trichodesmium*, nitrogen fixation, phosphorus limitation, phosphorus use efficiency, climate change

## Abstract

Surface temperature in the ocean is projected to be elevated and more variable in the future, which will interact with other environmental changes like reduced nutrient supplies. To explore these multiple stressor relationships, we tested the influence of thermal variation on the key marine diazotrophic cyanobacterium *Trichodesmium erythraeum* GBRTRLI101 as a function of the limiting nutrient phosphorus (P). Two constant temperature treatments represented current winter (22°C) and summer (30°C) mean values. Three variable temperature treatments fluctuated around the constant control values: Mean 22°C, either ± 2°C or ± 4°C; and mean 30°C ± 2°C. Each thermal treatment was grown under both P-replete (10 μmol/L) and P-limiting conditions (0.2 μmol/L). Effects of thermal variability on *Trichodesmium* were mainly found in the two winter variable temperature treatments (22°C ± 2°C or ± 4°C). P availability affected growth and physiology in all treatments and had significant interactions with temperature. P-replete cultures had higher growth and nitrogen and carbon fixation rates in the 22°C constant control, than in the corresponding variable treatments. However, physiological rates were not different in the P-replete constant and variable treatments at 30°C. In contrast, in P-limited cultures an advantage of constant temperature over variable temperature was not apparent. Phosphorus use efficiencies (PUE, mol N or C fixed h^-1^ mol cellular P^-1^) for nitrogen and carbon fixation were significantly elevated under P-limited conditions, and increased with temperature from 22 to 30°C, implying a potential advantage in a future warmer, P-limited environment. Taken together, these results imply that future increasing temperature and greater thermal variability could have significant feedback interactions with the projected intensification of P-limitation of marine N_2_-fixing cyanobacteria.

## Introduction

All environmental factors that affect the growth and fitness of organisms vary over time, and these biological impacts are projected to intensify as environmental variability increases under global climate change scenarios (Burroughs, 2007; IPCC, 2012; Thornton et al., 2014; Boyd et al., 2016). In particular, sea surface temperature fluctuations are mainly driven by air-sea heat exchange and short-wave radiation penetration (Schneider and Zhu, 1998; Hazeleger and Haarsma, 2005; Hosoda et al., 2016). In a more stratified warming ocean, an expected shallower upper mixed layer will become more vulnerable to disturbance and more sensitive to varying air-sea heat fluxes and solar radiation (Behrenfeld et al., 2006; Häder et al., 2011; Boyd et al., 2016). Consequently, thermal variability is predicted to increase in the future surface ocean (IPCC, 2012).

Global warming effects have been addressed in voluminous studies, but until recently the influence of changing thermal variability on ecosystems has received relatively little attention. Several recent studies in terrestrial ecosystems examined the diverse impacts of thermal variation on ectothermic animals (Bozinovic et al., 2011; Estay et al., 2011; Paaijmans et al., 2013) or soil bacterial communities (Langenheder et al., 2012). The biological effects of thermal variability have also been examined in marine metazoans (Dong et al., 2006; Tian and Dong, 2006; Putnam and Edmunds, 2011; Godbold and Solan, 2013). For phytoplankton, Bernhardt et al. (2018) proposed a non-linear averaging model based on the principle of Jensen’s inequality, suggesting that temperature fluctuations exceeding the thermal optimum can depress temperature growth response curves compared to the constant temperature condition.

Most investigations of thermal effects on marine phytoplankton, however, have used constant experimental temperatures (Nishikawa and Yamaguchi, 2008; Fu et al., 2014; Sommer et al., 2016; Jiang et al., 2018; Sett et al., 2018), thus neglecting all natural temperature fluctuations from diel to seasonal timescales. This use of oversimplified thermal conditions in experimental design has the potential to lead to biased conclusions and problematic predictions. Thus, there is a need to investigate the impacts of thermal variability on important phytoplankton groups, especially under climate change scenarios. In this study, we explored the influence of thermal variation on the growth and physiology of the diazotrophic cyanobacterium *Trichodesmium erythraeum* GBRTRLI101.

As a dominant, globally distributed nitrogen-fixer in the tropical-subtropical oligotrophic ocean (Capone et al., 1997; Karl et al., 1997; Bell et al., 1999), *Trichodesmium* contributes a substantial fraction of marine biological nitrogen fixation and so in turn plays a key role in global nitrogen and carbon cycling (Karl et al., 2002; Capone et al., 2005; Bergman et al., 2013). The strain GBRTRLI101 used in this study was isolated in the Northern Great Barrier Reef (GBR) Lagoon (Fu and Bell, 2003), where frequent *Trichodesmium* blooms have been observed (Bell et al., 1999). The annual average sea surface temperature (SST) in this region is 26–27°C (De’ath et al., 2009) and the annual temperature range is approximately 4°C (Lough, 2007), with a seasonal SST range of 22–24°C in the winter and 28–32°C in the summer (Lough, 1998). This relatively stable SST provides optimal conditions for the growth of GBRTRLI101. Fu et al. (2014) determined that the optimal growth range of this strain is 24–28°C, coincidental with the average ambient temperatures. This culture isolate is presumably well-adapted to the relatively stable temperature of the tropical open ocean, and thus potentially sensitive to rapid thermal variation.

The growth of *Trichodesmium* in the northern GBR region also benefits from a relatively high ambient inorganic phosphate concentration (0.08–0.12 μmol/L) (Furnas, 1997). As a constitutive element in nucleotides, phospholipids, certain coenzymes and other important molecules in phytoplankton cells (Geider and La Roche, 2002; Sterner and James, 2002; Sohm et al., 2011), P plays important roles in cell structure, genetic information storage, gene expression, energy generation, protein synthesis and regulation of metabolic processes and signal pathways of cells (Sohm et al., 2011; Karl, 2014; Lin et al., 2016). The scarcity of P in the open ocean can lead to growth limitation of diazotrophic cyanobacteria (Sañudo-Wilhelmy et al., 2001; Ammerman et al., 2003; Fu and Bell, 2003), or to co-limitation with a concurrent deficiency of iron (Mills et al., 2004; Garcia et al., 2015; Walworth et al., 2016). Effects of P availability on the nitrogen fixation and growth of *Trichodesmium* GBRTRLI101 were investigated in previous studies (Fu and Bell, 2003; Fu et al., 2005).

The GBR region faces multiple climate change stressors, but ocean warming is notably problematic and is predicted to escalate in the future. The average SSTs of the GBR area in 1976–2005 were 0.4°C warmer than during the last three decades of the 19th century (Lough, 2007). By the end of this century, a 1–3°C average SST increase is projected to occur in this region, with the largest temperature increases in winter (Nakicenovic et al., 2000; Lough, 2007). More warm SST extremes and fewer cold SST extremes are also predicted to occur in the future GBR area (Lough, 2007). Understanding the differing effects of changing temperature magnitudes and amplitudes requires the use of experimental designs that can explore these aspects of intensive temperature variability.

Moreover, the strengthened stratification of the water column caused by future warming is predicted to sequentially reduce nutrient supplies from the deep ocean (Behrenfeld et al., 2006; IPCC, 2013). Bioavailable phosphorous in the surface ocean is thus likely to decrease, since it is mainly supplied to the sea surface by mixing and advective processes from the interior ocean (Karl, 2014). Consequently, the growth of *Trichodesmium* may be further stressed by P-limitation. The net effects of potential interactions between increasing thermal variability and decreasing P availability are, however, unknown.

We hypothesized that: (1) Thermal variability will have adverse impacts on the growth and physiology of *Trichodesmium* GBRTRLI101, and increasing the intensity of thermal variation will magnify these adverse influences, (2) P limitation will play a bigger role in stressing *Trichodesmium* growth than temperature variability; and (3) P-limited and P-replete cells will interact with temperature variation differently, with distinct consequences for physiology and growth. To test these hypotheses, we measured the growth rate, carbon fixation rate and nitrogen fixation rate, elemental stoichiometry, and Chl *a* to C ratio as physiological proxies of *Trichodesmium* GBRTRLI101 fitness. Finally, phosphorus use efficiency (PUE) for nitrogen fixation was calculated to quantitatively explore the interactive relationship between cellular phosphorus requirements and temperature.

## Materials and Methods

### Cultures and Incubation Methods

The *Trichodesmium* strain GBRTRLI101 used in this study was collected from waters close to the Low Isles (16°23′S,145°34′E) in the Northern Great Barrier Reef Lagoon (Fu and Bell, 2003). Cultures were maintained with autoclaved artificial seawater with iron, EDTA, trace metals and vitamins added as in the Aquil recipe (Sunda et al., 2005). All nutrients, metals and vitamins were 0.2 μm syringe-filtered before being added to the medium. Based on the previous study of Fu et al. (2005) on the relationship between inorganic P and growth of GBRTRLI101, 10 μmol/L of phosphate was used to maintain laboratory stock cultures. This concentration was also selected for the P-replete experimental condition in our study, while 0.2 μmol/L of phosphate was used for the P-limited condition. Before starting experiments using P-limited treatments, the P-replete stock cultures were acclimated to the phosphate concentration of 0.2 μmol/L for ∼3 weeks, until they achieved consistent P-limited growth rates. Cool white fluorescent bulbs were used to provide a 12 h dark: 12 h light cycle at 150 μmol photons m^-2^s^-1^. Cultures were grown in acid-washed 120-ml plastic jars fitting into an aluminum thermal block that provided an even temperature gradient, with uniform irradiance for all replicates. Constant temperature treatments were maintained at the same location within the thermal gradient throughout the experiments, while variable temperature treatments were achieved by manually switching the culture jars between two different temperatures every 2 days (see below).

The thermal performance curves (TPC) of GBRTRLI101 strain were determined across a temperature range from 16 to 36°C at intervals of 2°C, under both P-replete and P-limited conditions. Semi-continuous incubation methods were employed for 16 days, as described in Qu et al. (2018) and below. Cultures were diluted every 4 days. The initial biomass of all treatments for each cycle was carefully controlled to be low and invariant, at ∼100 μmol/L of particulate organic carbon (POC), and cultures were in early exponential growth stage, allowing the cultures to maintain exponential growth during the whole dilution cycle. To achieve this objective, Chl *a in vivo* fluorescence readings were measured with a Turner Designs 10-AU^TM^ fluorometer at both the beginning and end of the growth cycle to obtain the initial and final biomass of each cycle and determine the volume of the next dilution. Growth rates and nitrogen fixation rates were measured for all the temperature points in the thermal range, as described in *Physiological and Biogeochemical Analyses* below.

Based on the thermal limits and optimal range of GBRTRLI101 we obtained from the TPC experiment, and the SST records in the GBR area (Lough, 1998, 2007), we designed the constant and variable temperature treatments as shown in Supplementary Table S1. Two constant temperatures of 22°C and 30°C represented the mean values for “winter” (cool) and “summer” (warm) periods, respectively. For each constant temperature, one or two variable treatments were used simultaneously, each with an average temperature equal to the corresponding constant temperature. An “intense” 22 ± 4°C and a “mild” 22 ± 2°C variable treatment were included for comparison to the constant 22°C treatment. For 30°C, only one variable treatment 30 ± 2°C was used. The “intense” 30 ± 4°C treatment was not performed because the 34°C maximum temperature would have exceeded the maximum thermal tolerance of GBRTRLI101, especially under P-limited conditions. In each 4-day cycle, the first 48 h of variable treatments were at the lower temperature phase (LT phase, respectively, at 18, 20, and 28°C), and the second 48 h were at the higher temperature phase (HT phase, respectively, at 26, 24, and 32°C). Triplicate cultures were grown at both P levels for each constant and variable temperature treatment in order to investigate the interactions between phosphate availability and thermal variation. For thermal variation experiments semi-continuous incubation methods were employed in the same way as in the TPC determination experiment described above, except that dilutions of all temperature treatments were conducted between 4-day thermal cycles.

A steady state was reached after 15–16 cycles, at which point sampling began for specific growth rates, nitrogen and carbon fixation rates, Chlorophyll *a* content, and elemental stoichiometry. There were three sampling points in a growth cycle: the initial point (immediately after the dilution and before transferring cultures to the LT phase), the midpoint (48 h after the dilution, at the end of LT phase) and the final point (96 h after the dilution, at the end of HT phase). For variable temperature treatments, the data collected at the middle and final points were averaged as the mean value to compare with the data from the constant treatments.

### Physiological and Biogeochemical Analyses

#### Growth Rates

Specific growth rates (μ) of each replicate were calculated as in Qu et al. (2018), and the growth rate for each treatment was calculated as the mean growth rate of the triplicate bottles in each treatment. In view of the possible influence of temperature on Chl *a in vivo* fluorescence, only the *in vivo* readings at the initial and final points of each cycle, which were measured at the same temperature, were used to calculate the growth rates of each cycle. The *in vivo* readings collected at the middle point were only used to roughly estimate the growth of cultures in real time, but were not employed in the subsequent analyses. *In vivo-*derived specific growth rates were subsequently validated by particulate organic carbon/nitrogen (POC/PON) measurements.

#### Nitrogen Fixation Measurements

N_2_-fixation rate was determined using the acetylene reduction method (Capone, 1993) with a gas chromatograph GC-8a (Shimadzu Scientific Instruments). For all treatments, 10 ml of culture from each replicate was transferred to a 27 ml serum vial, which was immediately sealed. Afterward, 2 ml of air was extracted while 2 ml of acetylene was injected with a syringe to the headspace of each serum vial. A theoretical 3:1 ratio (mol acetylene to mol N_2_ reduced) was used to calculate the N_2_ fixation rates based on the rates of ethylene production (Montoya et al., 1996). After 4 h incubation (from 11:00 am to 15:00 pm) during the light period (Tuit et al., 2004), ethylene production was measured by injecting 200 μl of headspace to the GC-8a device and comparing the reading to that of the same amount of a 100 ppm ethylene standard (GMT10325TC, Matheson Gas Products). The raw N_2_-fixation rates were normalized to the PON concentration of each sample. In the calculation of PUE, raw N_2_-fixation rates were normalized to cellular P content measured as particulate organic phosphorus (POP).

#### C Fixation Rates

Photosynthetic carbon fixation rates were measured using the ^14^C-uptake technique. 50 nCi ^14^C-NaH^14^CO_3_ (PerkinElmer, Inc.) was added to 10 ml subsamples of each replicate, resulting in a final specific activity of ∼0.185 kBq/ml. Dissolved inorganic carbon in the background culture was determined as in Qu et al. (2018). As with the nitrogen fixation measurements, all subsamples were incubated for 4 h (11:00 to 15:00) in the middle of the light period, and then filtered onto GF/F filters. Each filter was then placed in 5 ml scintillation fluid in a 7 ml scintillation vial overnight. Triplicated total radioactivity (T_A_) samples and blanks of each treatment were prepared following the same protocol in Qu et al. (2018). ^14^C radioactivity was determined using liquid scintillation counting (PerkinElmer, Inc.) for T_A_, blanks and culture samples.

#### Elemental Stoichiometry

Elemental ratios were calculated based on the measurements of POC, PON, and POP. 30 ml of culture was filtered onto a pre-combusted (500°C, 2–3 h) GF/F filter and dried at 55–65°C for POC and PON analysis. POC and PON were determined with a Costech Elemental Analyzer using methionine and acetanilide standard for standard curves. Blank GF/F filters were used to measure the background carbon in the filter (Qu et al., 2018). Another 20 ml aliquot from each replicate was filtered through pre-combusted GF/F filters and prepared for POP measurements following the protocol described in Qu et al. (2018). The standard molybdate colorimetric method was applied in the analysis of the POP samples (Solorzano and Sharp, 1980). Blank GF/F filters were treated as samples to determine background phosphorous contents.

#### Chlorophyll *a* (Chl *a*)

As an important indicator of photo-acclimation and chlorophyll-specific biomass in phytoplankton (Cloern et al., 1995; Geider et al., 1997), Chl *a* was extracted from *Trichodesmium* cultures. 20 ml of culture from each replicate was filtered onto GF/F glass fiber filters, which were then extracted in 6 ml of 90% acetone for 24 h at -20°C. The non-acidification method was employed to measure Chl *a* content with a Turner Designs 10-AU^TM^ fluorometer (Welschmeyer, 1994). Chl *a* content was normalized to POC to test for changes in the Chl *a*: carbon ratios in the treatments, which is a commonly used proxy of growth, productivity and photosynthetic capacity of phytoplankton (Cloern et al., 1995; Geider et al., 1997; Sathyendranath et al., 2009; Jakobsen and Markager, 2016).

### Statistics

Based on the actual growth rates and nitrogen fixation rates at constant temperature points from 16 to 36°C, the thermal performance curves (TPC) were calculated from the model of Norberg (2004) and Thomas et al. (2012). To make predictions about the effects of temperatures fluctuating every 2 days on a magnitude of ± 2 or ± 4°C, a non-linear averaging model that takes the principle of Jensen’s inequality into account was employed (Bernhardt et al., 2018). This model is based on the time cells spend at each portion of their TPC during thermal fluctuations and incorporates the observation that growth rates typically decline much more precipitously at the warm end of the TPC compared to the cool end. Unlike previous models applying an unrealistic linear relationship between temperature and population growth, this non-linear averaging model provides quantitative and accurate estimates of the growth and persistence of phytoplankton species in a thermally variable environment. It was tested with the green alga *Tetraselmis tetrahele* under light and nutrient-saturated conditions, and provides useful projections such as critical threshold temperatures for phytoplankton growth (Bernhardt et al., 2018).

To test the interactive effects between thermal variation and P availability, Univariate Analysis of Variance (two-way ANOVA) was carried out with R v3.3.1, while one-way ANOVA was used to analyze the difference among temperature treatments for both P conditions. The Tukey multiple comparison test was applied for multiple comparisons between individual treatments once significant difference was observed by ANOVA. Student’s *t*-test was employed when comparing the difference between constant 30°C and its variable 30 ± 2°C treatment at both P concentrations. All significance testing was done at the *p*-value < 0.05 level.

## Results

### Thermal Performance Curves (TPC) for Growth and Nitrogen Fixation Rates

**FIGURE 1:**
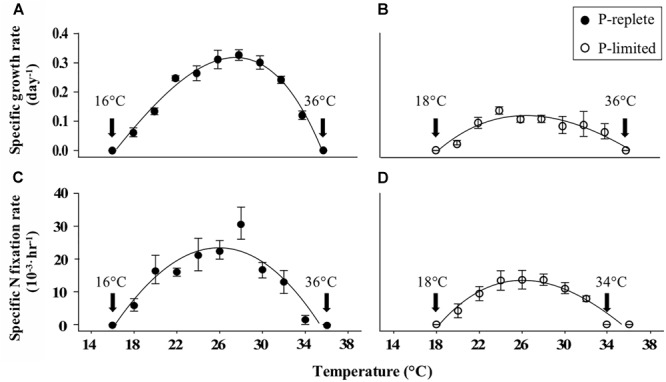
Measured thermal response curves (TPC) based on growth rates of *Trichodesmium erythraeum* strain GBRTRLI101 under **(A)** P-replete (solid symbols) and **(B)** P-limited (open symbols) conditions. Nitrogen fixation rates normalized to PON are shown in **(C)** P-replete and **(D)** P-limited cultures at nine temperatures. Values and error bars represent the means and the standard deviations of triplicate cell cultures in each treatment. The arrows labeled with temperature values show the temperatures where growth or N_2_ fixation was not detectable.

Incorporating the measured growth rates at constant temperatures from 16 to 36°C into the thermal response model (Norberg, 2004; Thomas et al., 2012) yielded the TPCs under P-replete (Figure 1A) and P-limited (Figure 1B) conditions, which showed an expected “increase-peak-decline” pattern. As expected, P limitation substantially decreased both nitrogen fixation rates and growth rates. The optimal growth temperature (T-opt) for both TPCs at constant temperatures under both P conditions was ∼27°C (Table 1). No growth was observed at either the coldest (16°C) or warmest (36°C) temperatures tested, regardless of the P concentration. The maximum growth rates were 0.32 and 0.12 d^-1^, respectively, for P-replete and P-limited cultures in the TPCs. P-limitation also narrowed the width of the thermal niche by ∼2°C (minimum to maximum limits) compared to the P-replete condition (Figure 1A,B).

**Table 1:** Thermal performance curve (TPC) parameters at constant and two variable temperature treatments (±2 and ±4°C), all at two P concentrations (10 and 0.2 μmol/L).

Phosphate status	TPC type	T-max	T-min	T-opt	T-width	μ_max_	*R*^2^
P-replete	Constant temp	35.9	16.3	27.7	19.6	0.32	0.97
	Variable ±2°C	35.6	16.3	27.5	19.3	0.30	0.93
	Variable ±4°C	34.5	16.6	26.8	17.9	0.26	N.A.
P-limited	Constant temp	36.6	18.2	26.3	18.4	0.12	0.75
	Variable ±2°C	36.5	18.5	26.5	18	0.12	0.70
	Variable ±4°C	36	19.6	27.0	16.4	0.10	N.A.

*Values in the table were calculated by incorporating the measured growth rates at constant temperatures (16–36°C) and variable temperature treatments (22 ± 2°C, 22 ± 4°C, 30 ± 2°C) into the thermal response model of Norberg (2004) and Thomas et al. (2012). T-max, upper temperature limit; T-min, lower temperature limit; T-opt, optimal temperature; T-width, temperature range; μ_*max*_, maximal growth rate; *R*^*2*^, the correlation factor, in the range of 0∼1. The closer *R*^*2*^ is to 1, the better the model-predicted growth rates match the actual measured ones. Since only one ±4°C temperature treatment was conducted, the *R*^*2*^ for it is not available (N.A.).*

The TPCs based on nitrogen fixation rates at the same constant temperature range of 16–36°C (Figure 1C,D) were consistent with the TPCs of growth rates. The highest nitrogen fixation rates across the TPC corresponded with the optimal temperature for growth (24–30°C). Moreover, with sufficient P supply GBRTRLI101 could survive and fix nitrogen at 18°C, while at the same temperature the growth and nitrogen fixation in P-limited cultures stopped. Although both P-replete and P-limited cultures showed slight growth at 34°C, the measured nitrogen fixation rates under P limitation were close to zero.

**FIGURE 2:**
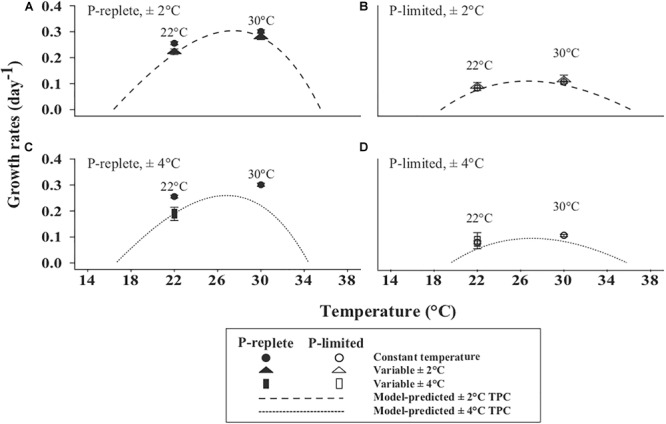
Non-linear averaging model-predicted TPCs for the ±2°C (dashed line) **(A,B)** and ±4°C (dotted line) **(C,D)** treatments. **(A,C)** are under P-replete conditions and **(B,D)** under P-limited status. The actual measured growth rates of constant 22 and 30°C (solid cycle: P-replete; open circle: P-limited), ±2°C (solid triangle: P-replete; open triangle: P-limited) and ±4°C (solid rectangle: P-replete; open rectangle: P-limited) variable treatments are shown.

The non-linear averaging model-predicted TPCs under thermally variable conditions of ±2 and ±4°C (Bernhardt et al., 2018) are shown in Figure 2. In general, the model-predicted TPCs, especially with the magnitude of ±4°C, were narrowed and flattened compared to the corresponding TPCs based on measured growth rates at constant temperatures (Table 1). The comparisons between model-predicted TPCs and actually measured growth rates at constant and variable temperature conditions are presented in the following section.

### Growth Rates of Constant and Variable Temperature Treatments

Two-way ANOVA testing of the effects of temperature treatments, P concentrations, and their interactions on the measured growth rates of strain GBRTRLI101 in both seasons showed that temperature variations played a minor role (*p*-value > 0.05). In contrast, the impacts of P conditions and the interactions between temperature and P condition were significant (*p*-value < 0.05). The effects of thermal fluctuation were further analyzed for two seasons and two P concentrations as follows.

For “winter” treatments, the measured growth of strain GBRTRLI101 showed different responses to thermal variation at the two P concentrations (Figure 2). Under P-replete conditions, the average growth rates in three temperature treatments were significantly different (one-way ANOVA, *p*-value < 0.05), with a sequential declining trend from constant 22°C to “mildly” and “intensely” variable temperature treatments. Specifically, Tukey multiple comparison showed that the growth rate of the variable 22 ± 4°C treatment was significantly lower than that of the constant 22°C treatment (Figure 2C, *p*-value < 0.05). At the growth-limiting P concentration, the declining trend driven by thermal variation relative to the constant 22°C treatment disappeared (Figure 2B,D, one-way ANOVA, *p*-value > 0.05).

Likewise, for constant 30°C and its variable treatment 30 ± 2°C, the adverse effect of thermal variation on the actually measured growth rate showed a smaller but still significant difference only in the P-replete condition (Figure 2A, Student’s *t*-test, *p*-value < 0.05). Under the P-limited condition, the measured growth rates at constant and variable temperature treatment showed no significant difference (Figure 2B, *p*-value > 0.05).

In the modeled TPC based on non-linear averaging (Bernhardt et al., 2018), temperature fluctuation was predicted to decrease the growth rate of GBRTRLI101 compared to the constant temperature condition, and more intense thermal variations were predicted to have a more profound adverse effect on growth (Figure 2). Under P-replete conditions, the measured growth rates in the 22 ± 2°C, 22 ± 4°C, and 30 ± 2°C variable treatments all closely fit the predicted curves (Figure 2A,C). In P-limited cultures, however, the measured growth rates in the 22 ± 2°C, 22 ± 4°C, and 30 ± 2°C variable treatments did not respond to the thermal variability of ±2°C (Figure 2B) and ±4°C (Figure 2D) as predicted by the model and showed similar growth rates at constant and variable temperature treatments.

### Nitrogen and Carbon Fixation Rates in Thermal Variation Experiments

**FIGURE 3:**
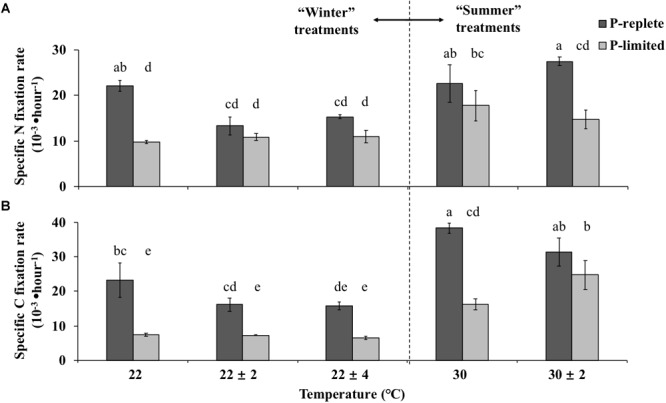
Average **(A)** nitrogen-specific fixation rates and **(B)** carbon-specific fixation rates normalized to PON and POC, respectively, of *Trichodesmium* GBRTRLI101 in five constant and variable temperature treatments under two P conditions (10 and 0.2 μmol/L) in summer and winter experiments. Values and error bars, respectively, represent the means and the standard deviations of triplicate cell cultures under each treatment. The letters represent the significant differences based on the grouping results of the Tukey multiple comparison among ten temperature ×P treatments.

Nitrogen and carbon fixation rates of GBRTRLI101 were also driven by the interactive effects of temperature fluctuation and P concentration (Figure 3A,B). For “winter” temperature treatments, the average nitrogen fixation rates of GBRTRLI101 in both 22 ± 2°C and 22 ± 4°C treatments were significantly lower than in the constant 22°C treatment (one-way ANOVA and Tukey multiple comparison, *p*-value < 0.05) under P-replete conditions (Figure 3A). The average carbon fixation rates of P-replete cultures in variable treatments showed a slight decrease, compared to the rate at constant 22°C (one-way ANOVA, *p*-value > 0.05) (Figure 3B). In P-limited cultures, however, the advantage of constant 22°C temperature treatment in both carbon and nitrogen fixation over the corresponding variable temperature treatments disappeared (*p*-value > 0.05).

For the summertime treatment, the average nitrogen and carbon fixation rate showed no significant difference between constant 30°C and variable 30 ± 2°C treatment at P-replete status (Figure 3A,B, Student’s *t*-test, *p*-value > 0.05). However, under the P-limiting condition, the carbon fixation rate at variable 30 ± 2°C was significantly higher than at constant 30°C (Figure 3B, Student’s *t*-test, *p*-value < 0.05). No difference was found for the nitrogen fixation rate (*p*-value > 0.05).

### Elemental Stoichiometry and Chl *a* to Carbon Ratio

Cellular C: N, C: P, and N: P ratios (mol to mol) based on POC, PON, and POP measurements changed with the two P levels in both “winter” and “summer” treatments (Table 2, two-way ANOVA, *p*-value < 0.05). As expected, P limitation dramatically increased C: P and N: P ratios at all temperature treatments. For all the three elemental ratios, though, the effects of temperature fluctuation and the interaction between P conditions and thermal variation were minor (Table 2, *p*-value > 0.05).

**Table 2:** Elemental stoichiometry in five constant and variable temperature treatments at two P concentrations (10 and 0.2 μmol/L) in summer and winter experiments.

	P-replete			P-limited		
*“Winter” treatment*	C: N	C: P	N: P	C: N	C: P	N: P
Average constant 22°C	6.70 ± 0.59	106.48 ± 1.98	15.86 ± 0.51	6.98 ± 0.81	350.91 ± 65.80	46.30 ± 13.85
Average 22 ± 2°C	7.40 ± 0.47	126.09 ± 10.91	17.04 ± 1.34	8.09 ± 0.57	342.08 ± 28.75	42.12 ± 4.15
Average 22 ± 4°C	7.18 ± 0.44	125.90 ± 4.91^∗^	18.73 ± 0.55^∗^	7.66 ± 0.36	342.09 ± 21.96	44.59 ± 1.53

***“Summer” treatment***	**C: N**	**C: P**	**N: P**	**C: N**	**C: P**	**N: P**

Average constant 30°C	6.76 ± 0.66	116.99 ± 15.87	18.02 ± 1.20	7.35 ± 0.58	317.66 ± 15.73	42.94 ± 1.01
Average 30 ± 2°C	6.30 ± 0.55	115.94 ± 5.26	18.44 ± 0.71	7.10 ± 1.02	324.26 ± 21.96	45.15 ± 1.53

*The values in the table are the average values of triplicates ± standard deviations. The asterisks show the significant differences between the variable temperature treatment and the corresponding constant temperature treatment.*

In the P-replete “winter” treatment groups, C: N, C: P, and N: P ratios at constant 22°C were consistent with the Redfield Ratio (Redfield, 1958). The C: N at variable treatments was slightly higher than the ratio at constant 22°C for both P concentrations (one-way ANOVA, *p*-value > 0.05). The P-replete C: P and N: P ratios in the 22 ± 4°C treatment were significantly higher than in the constant 22°C treatment (Tukey multiple comparison, *p*-value < 0.05), but none of the three elemental ratios were significantly different between the constant 22°C and the two variable treatments in the P-limited cultures (Table 2). In the P-replete “summer” treatment groups, elemental ratios of all three thermal treatments were close to the Redfield Ratio, and no significant differences were observed at either P level (Table 2, *p*-value > 0.05). The elemental ratios during the high and low phases of the variable temperature treatments are shown in Supplementary Table S2.

**FIGURE 4:**
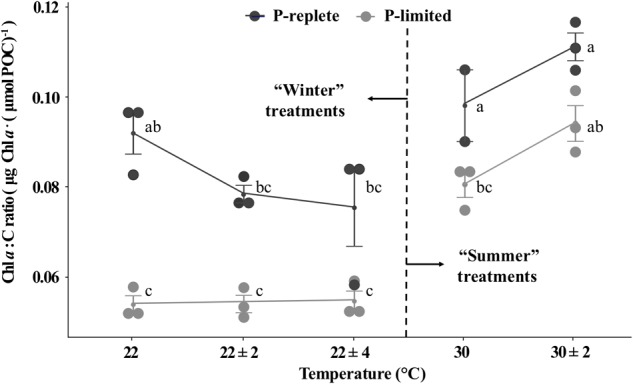
Chl *a* to C ratios of *Trichodesmium* GBRTRLI101 at five constant and variable temperature treatments and two P conditions (light gray symbols: P-limited culture, 0.2 μmol/L; dark gray symbols: P-replete culture, 10 μmol/L) in summer and winter experiments. Values and error bars, respectively, represent the means and the standard deviations of triplicate cell cultures under each treatment. The letters represent the significant differences based on the grouping results of the Tukey multiple comparison among ten temperature × P treatments.

The P-replete Chl *a* to carbon ratios (g : mol) were significantly higher than ratios under P-limitation for both “winter” and “summer” time (Figure 4, two-way ANOVA, *p*-value < 0.05). However, due to the relatively large error bars, neither temperature variation individually nor the interactions between temperature and P conditions significantly changed the Chl *a* to C ratios (two-way ANOVA, *p*-value > 0.05). Moreover, different responses to thermal variation were observed for “winter” and “summer” treatments and the role of P concentrations varied for the two seasons (Figure 4). In the low temperature treatments, thermal variability slightly decreased the P-replete Chl *a* to C ratios, while P-limited cultures did not respond to thermal variation (one-way ANOVA, *p*-value > 0.05). In summertime, temperature variation slightly lifted the Chl *a* to C ratio for both P conditions (Student’s *t*-test, *p*-value > 0.05). These patterns were similar to the responses of nitrogen-fixation to temperature variation (Figure 4).

### Phosphorous Use Efficiency (PUE)

**FIGURE 5:**
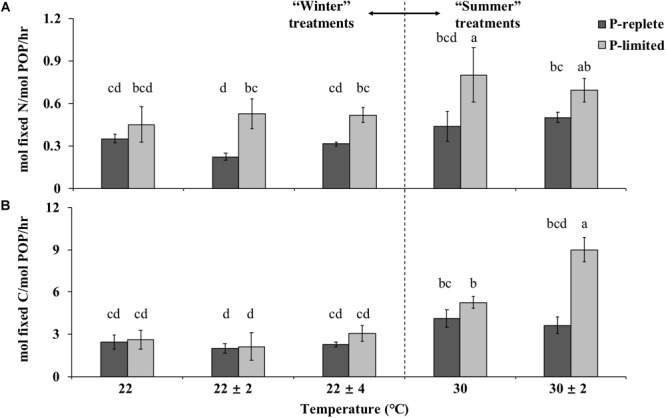
Phosphate Use Efficiency (PUE) for nitrogen **(A)** and carbon **(B)** fixation under P-replete and P-limited conditions (10 and 0.2 μmol/L) in five different constant and variable temperature treatments in summer and winter experiments. Values and error bars, respectively, represent the means and the standard deviations of triplicate cell cultures under each treatment. The letters represent the significant differences based on the grouping results of the Tukey multiple comparison among ten temperature × P treatments.

The PUE for nitrogen and carbon fixation at different temperatures was determined by the amount of N or C fixed per unit time (mol h^-1^) per unit cellular P (mol^-1^), respectively, named as PUE_N_ and PUE_C_ here. P availability, temperature treatment and their interactions all played significant roles in determining the PUE of cultures (Figure 5, two-way ANOVA, *p*-value < 0.05).

In both “summer” and “winter” treatments, PUE_N_ values in P-limited cultures were significantly higher than under P-replete conditions (two-way ANOVA, *p* < 0.05; Figure 5A). In the “winter” treatment group, PUE_N_ values of the three thermal treatments were significantly different under P-replete conditions (one-way ANOVA, *p*-value < 0.05). The P-replete PUE_N_ of the constant 22°C treatment was the highest, followed by the 22 ± 4°C treatment and then the 22 ± 2°C treatment. The difference between 22 ± 2°C and the other two treatments was significant (*p*-value < 0.05). The higher PUE_N_ at constant 22°C compared to the two variable temperature treatments was consistent with the higher growth rate at constant temperature. However, this pattern was not observed in P-limited “winter” treatments, or in the “summer” treatments with either of the two P conditions (Figure 5A, *p*-value > 0.05).

In most temperature treatments, PUE_C_ in P-limited cultures was only slightly higher than in the cultures with sufficient P, except at 30 ± 2°C where the difference in PUE_C_ between the two P concentrations was significant (Figure 5B, *p*-value < 0.05). Moreover, under P limitation, the PUE_C_ at 30 ± 2°C greatly exceeded the value at constant 30°C (*p*-value < 0.05), consistent with the pattern for carbon fixation rates (normalized to POC) in these treatments. In addition, under both P-replete and P-limited conditions and both constant and variable temperatures, the PUE for both nitrogen or carbon fixation increased when temperature rose from 22 to 30°C. This increase of PUE with rising temperature was especially significant for carbon fixation at both P concentrations (Figure 5B, Student’s *t*-test, *p* < 0.05).

## Discussion

Our study examined the individual effects of temperature variability and P availability and their interactions, using temperature treatments representative of two seasons. Temperature variability affected growth, nitrogen and carbon fixation rates and elemental ratios differently compared to constant temperatures at the same mean value. This was especially evident in the “winter” treatments, suggesting that the responses of *Trichodesmium* to thermal variability may vary in different seasons or in different parts of their latitudinal temperature range. These results thus partly supported our first hypothesis that thermal variability would have adverse impacts on *Trichodesmium* growth and physiology.

P limitation obviously decreased all the growth and physiological rates measured, and shifted the elemental ratios, under both “winter” and “summer” temperature treatments. As expected in our second hypothesis, these nutrient limitation effects were often larger individually than those of variable thermal regimes. Interactions between P levels and thermal variation were widely observed when comparing measured growth rates with the model-predicted TPCs. Interactions were also manifested through differences in nitrogen and carbon fixation rates, particularly in the three “winter” temperature treatments. This provided support for our third hypothesis, that P availability would affect the responses of *Trichodesmium* to thermal variability due to interactive effects.

### P Availability and Phosphorus Use Efficiency (PUE)

P limitation played a large role in narrowing the temperature range of the TPC, decreasing growth and fixation rates, and shifting the elemental ratios and Chl *a* content of *Trichodesmium* GBRTRLI101. Generally, the effects of P limitation were larger than the impacts of temperature variability, supporting our second hypothesis. In our study, growth and nitrogen fixation rates under P-limitation dropped down significantly compared to the corresponding temperature treatment in the P-replete condition. The importance of P in cellular structural components like membranes and other key biomolecules such as nucleic acids accounts for the severely stressed growth and metabolic rates of phytoplankton cells under P limitation (Toby, 1999; Ji and Sherrell, 2008; Liu et al., 2011). Specifically, the negative effects of P deficiency on nitrogen fixation in *Trichodesmium* have been reported by numerous studies (Sañudo-Wilhelmy et al., 2001; Fu et al., 2005; Hynes et al., 2009; Orchard et al., 2009).

It is not surprising that a large increase in the C: P and N: P ratios relative to the Redfield Ratio (Redfield, 1958) was observed under P limitation, since the cellular P content decreased relative to other elements. Moreover, P limitation also significantly decreased the Chl *a* to C ratio. An increasing cellular C: P ratio was found to give rise to a decrease in the Chl *a* to C ratio in marine phytoplankton under nutrient limitation (Tett et al., 1975), in accordance with our results. Guildford and Hecky (2000) also extrapolated a universal positive correlation between Chl *a* abundance and P availability. The decreased Chl *a* to C ratio and the concurrent reduced carbon fixation rate under conditions of P limitation both implied a decline in photosynthetic capacity.

The positive correlation between PUE and nitrogen or carbon fixation rates at constant and variable temperature in our study indicated that PUE is a useful parameter to evaluate the potential capacity of *Trichodesmium* cells to survive and grow in a P-stressed environment. A major increase in PUE under P-limited conditions was observed when compared to P-replete conditions. This may be at least partly attributed to the luxury uptake of P under sufficient P availability, leading to excess intracellular P storage and hence a lower PUE (Droop, 1973; Ducobu et al., 1998; Lin et al., 2016). Thus, intracellular P used in our calculation of PUE in P replete cultures likely included not only P actively involved in cell structure and functions, but also stored P, which may lead to a minimum estimate of marginal PUE. However, within the same temperature range the pattern of PUE increasing with temperature was observed under both P-replete and P-limited conditions, suggesting that PUE could be still be used as a proxy in environmental surveys, although interpretations will need to be made in the context of *in situ* P availability. It is also notable that after several growth cycles under P limitation, the stored P in our cultures had been consumed to support growth, nitrogen fixation and other cell activities. In this sense, our results imply considerable flexibility in P storage and utilization in *Trichodesmium* cells.

Elevated iron and phosphorous use efficiencies with temperature increase have also been observed in both Fe-replete and Fe-limited cultures of the Atlantic isolate *Trichodesmium erythraeum* IMS101 (Jiang et al., 2018). This suggests a commonly occurring positive correlation between Fe or P use efficiency and temperature in *Trichodesmium*. Elevated intracellular metabolic rates at high temperature within the thermal limits of phytoplankton (Goldman and Carpenter, 1974; Goldman, 1979) could thus allow for higher growth and nitrogen fixation rates supported by less cellular P and Fe (Jiang et al., 2018).

Another possible interpretation is varying nutrient allocation at different temperatures. Toseland et al. (2013) found that phytoplankton require less cellular P relative to N at high temperatures, as faster protein synthesis rates decrease the numbers of phosphorus-rich ribosomes and associated rRNAs required to support growth. Thus, the combination of up-regulated metabolic rates and a reduced P requirement in ribosome synthesis may explain elevated PUE values at warmer temperatures. However, the hypothesis of Toseland et al. (2013) needs to be further tested for *Trichodesmium* by measuring the ribosomal content of cells grown at different temperatures. Clearly though, in the future warming ocean increased PUE at higher temperature may help *Trichodesmium* to persist under P-limited conditions.

### Impacts of Intensity of Temperature Variation

In the “winter” temperature treatments, we used “mildly” and “intensely” variable treatments to investigate the effects of intensity of thermal variation. For some physiological proxies, the intensity of thermal variation was found to modulate the responses of *Trichodesmium* to thermal variability and P concentrations. For instance, the growth rate under P-replete conditions sequentially decreased in 22 ± 2°C and 22 ± 4°C treatments, compared to the constant 22°C treatment. The statistically significant difference was between 22 and 22 ± 4°C, which indicated an enhancement effect of intensified temperature variability. A similar pattern was also observed in the N: P ratios of the three “winter” treatments under P replete conditions. Notably, under P limited conditions, the growth rate and elemental ratios were similar at variable 22 ± 2°C, 22 ± 4°C and constant 22°C treatments. These results imply that the effects of thermal fluctuation intensity are dependent on nutrient availability.

Marañón et al. (2014) indicated that in general the impact of nutrient availability on the growth of phytoplankton exceeds that of temperature. However, our observation that the growth rates at two P concentrations were close in variable 22 ± 4°C treatments suggests a comparable effect of “intensely” variable temperature with nutrient limitation on stressing the growth. In other words, the magnitude of the temperature fluctuation range matters when comparing effects of nutrient availability and thermal variation.

### Interactions Between TemperatureVariability and P Availability in TwoSeasons

Much previous research has indicated that until some upper threshold is reached, temperature increases generally promote the growth of nutrient-replete phytoplankton by stimulating their metabolic rates (Eppley, 1972; Goldman and Carpenter, 1974; Fu et al., 2014; Sherman et al., 2016). This trend is in agreement with our results from two TPCs at both P concentrations, and also with the growth rates of P-replete cultures at constant 22°C and 30°C. However, our study suggests that this trend becomes much more complicated when temperature fluctuates instead of simply rising, and also when nutrient availability is involved.

A comparison of our TPCs measured at two P concentrations shows that under P-limited conditions growth rates declined, and the survival and nitrogen fixation temperature range narrowed. In the marine diatom *Thalassiosira pseudonana*, the interaction between nutrient limitation and temperature was found to intensify vulnerability to warming. This is because growth-limiting conditions shifted the optimum temperature zone of the species toward the lower temperature end of the nutrient-replete TPC (Thomas et al., 2017). Fe-limited *Trichodesmium*, however, exhibit an opposite trend, in that their TPC is shifted to the right, toward warmer temperatures (Jiang et al., 2018). Temperature-nutrient interactions also played a large role in shaping the TPC and determining thermal limits in our *Trichodesmium* experiments. Specifically, the strain GBRTRLI101 became more vulnerable to extreme temperatures, with constrictions of both the upper and lower thermal limits under P limitation.

An evaluation of physiological responses to thermal variation under two distinct P conditions clearly revealed interactions between the two variables as well. Under P-replete conditions, temperature variation decreased the growth and nitrogen fixation rates of *Trichodesmium*, as predicted in our first hypothesis. This observation was consistent with the projection of the non-linear averaging model in Bernhardt et al. (2018). Under the P replete condition, the measured growth rates in the variable temperature treatments fit the model-predicted TPC well, supporting the validity of the model at least in the suboptimal and supraoptimal temperature range. Under P-limitation, however, the growth advantage of constant temperature treatments over variable temperature treatments became negligible. P-limited growth rates did not further decline at variable temperatures, and so deviated from the predicted curves, especially in the ±4°C treatment. The reason for this observation could be that when the Bernhardt et al. (2018) model was developed, experimental quantification in constant and fluctuating environments was only carried out under nutrient-saturated conditions. This suggests a limitation of the model under low nutrient availability.

Furthermore, as expected P-limited cultures showed consistently lower nitrogen and carbon fixation rates and very elevated N: P and C: P ratios. Consequently, these proxies remained statistically identical among the three “winter” temperature treatments. In contrast, significant responses to thermal variation were observed among the same three treatments under P-replete conditions.

Several recent studies focusing on the responses of phytoplankton to temperature under nutrient limiting conditions may help to explain the inconsistent effects of temperature variation at the distinct P conditions in our study. Marañón et al. (2018) discovered a lack of temperature sensitivity in the metabolic rates of three widely distributed and biogeochemically important phytoplankton species (*Synechococcus* sp., *Skeletonema costatum* and *Emiliania huxleyi*) under nitrogen-limitation, which was distinct from the results of nitrogen-replete treatments. They interpreted this result based on classical Monod or Michaelis-Menten enzyme kinetics, and attributed the minimal temperature dependence under N-limiting conditions to the similar temperature sensitivity of maximum reaction rate (*V*m) and half-saturation constant (*K*m) (Marañón et al., 2018). Thingstad and Aksnes (2019) further extended this explanation and concluded that the growth of nutrient-limited phytoplankton was limited by the molecular diffusion of extracellular nutrients, which is less temperature dependent compared to intracellular enzymatic processes. Jiang et al. (2018), however, suggested that rate limitation by micronutrient elements that are enzymatic co-factors, such as iron, may be much more responsive to temperature than nutrients that are major cellular structural components, such as N. In short, there is evidence that major nutrient limitation in particular may decrease the sensitivity of phytoplankton cells to temperature changes. This could help explain some of the less obvious effects of thermal variability on the physiology of *Trichodesmium* GBRTRLI101 under P limitation.

### Implications for *Trichodesmium* Growth and Biogeochemistry Under Climate Change

Under climate changes, our results suggest that the future trends of *Trichodesmium* GBRTRLI101 growth in the winter GBR area will depend on the combination of temperature increases and intensified thermal variability. Predictions of SST changes the GBR region during the winter season include 1–3°C warming and less cold extremes (Lough, 2007), which would seem to be beneficial to the growth of *Trichodesmium*. However, these climate changes are also likely to bring about potentially intensified thermal variability (Burroughs, 2007), which would adversely impact the growth and physiology of *Trichodesmium* cells especially in the winter, based on the results of this study.

In our study, 30 ± 2°C did not profoundly change the growth and physiology of *Trichodesmium* cells. However, according to the temperature norm of this strain obtained in this study and in previous research (Fu et al., 2014), summertime temperatures above 33–34°C would greatly decrease the growth rate. In the future, more warm extremes are predicted to occur in the GBR area, similar to those that have already occurred in northeast Australia during 2015–2016 (Wolanski et al., 2017). In the future, a sea surface temperature increase of 1–3°C is expected to occur in the GBR area (Nakicenovic et al., 2000; Lough, 2007), with the maximal seawater temperature exceeding the upper growth threshold of *Trichodesmium*. Moreover, even with only 1°C of warming, the number of summer days with a temperature higher than 33°C is projected to increase by 3–4 fold compared to the years from 1961 to 1990 (Lough, 2007). Under this scenario, *Trichodesmium* GBRTRLI101 may find it difficult to survive in future summers with more very warm days and episodic heatwaves.

Other *Trichodesmium* species and strains are widely distributed in the tropical and subtropical Pacific, North Atlantic and Indian Ocean (Capone et al., 1997, 2005; Sohm et al., 2011), where the expected trends of future warming, reduced nutrient supplies and increased thermal variability are similar to those of the GBR area (Beardall et al., 2009; IPCC, 2012; Lin et al., 2013; Häder et al., 2014; Basu and Mackey, 2018). In this context, the pattern we observed in this study could provide insights into the potential responses of other *Trichodesmium* strains in different ocean regions to climate changes. For instance, the current annual SST range of the tropical and subtropical Pacific Ocean is ∼23–30°C, with a diurnal thermal variability of 0.4–1.5°C observed in wintertime when the SST is ∼23.5°C (Clayson and Weitlich, 2007; Dunstan et al., 2018). This provides a comparable temperature background to our study examining the GBR area, and may result in similar growth declines of *Trichodesmium* in the winter if thermal variability is intensified in the future. On the other hand, *Trichodesmium* in the tropical Atlantic Ocean experience an annual temperature range of ∼26–29°C (Keenlyside and Latif, 2007; Muñoz et al., 2012) which resembles the summertime situation in the GBR region, possibly leading to only minor responses of *Trichodesmium* to thermal variability. However, more investigations will be needed to verify these extrapolations of our results to other ocean basins dominated by *Trichodesmium* species and strains with potentially different thermal adaptation histories.

Climate change is also predicted to decrease nutrient supplies to the euphotic zone of the ocean, and in turn the export of organic particles to the deep ocean (Hutchins and Fu, 2017). Under P-replete conditions, thermal variation had negative effects on most cellular parameters, while under P-limitation, the adverse impacts became negligible. As a result, the possible implications of temperature variability for future biogeochemistry should be considered to depend on P availability.

Adverse effects on *Trichodesmium* growth and nitrogen fixation and increased N: P and C: P ratios were especially evident under wintertime P-replete, thermally variable conditions. This trend was even more marked when the intensity of the temperature fluctuations increased. Hence, if P availability in the surface seawater of the GBR area is maintained at currently sufficient levels (Fu and Bell, 2003; Fu et al., 2005) and more intensified temperature variability occurs in winter time, the contribution of *Trichodesmium* GBRTRLI101 to primary production and new nitrogen supply would be reduced. In the meanwhile, more C and N relative to P might be exported to the deep ocean with ratios exceeding the Redfield Ratio. In summer, the growth of *Trichodesmium* GBRTRLI101 and related biogeochemistry would not be obviously changed by thermal variation, other than adverse effects of extreme heat wave events as discussed above.

If vertically advected P supplies are lower in the future as predicted, P deficiency in the GBR region would affect the growth and physiology of *Trichodesmium* GBRTRLI101 in a similar pattern in both winter and summer. All the physiological proxies of *Trichodesmium* GBRTRLI101 would be profoundly stressed, and C: P and N: P in sinking organic particles would dramatically increase while the effects of thermal variation would be comparatively minor, as observed in our study.

Today, P availability in the GBR area (0.08–0.12 μmol/L) is higher than in the North Atlantic gyre (0.2–1.0 nmol/L), southwest Pacific Ocean (<20 nmol/L) and eastern Mediterranean (below 2.0 nmol/L) (Furnas, 1997; Wu et al., 2000; Thingstad et al., 2005; Moutin et al., 2007). As a result, *Trichodesmium* in the GBR area may suffer less than in the other ocean regions from intensified P limitation in the future ocean. Based on our results, the growth of *Trichodesmium* in the GBR area is more likely to be adversely impacted by thermal variability, while *Trichodesmium* in other more P-limited regions may not respond as much to temperature variations. Our findings also suggest that because *Trichodesmium* in the GBR area are relatively P-replete, it will be easier to use non-linear averaging models such the one we employed from Bernhardt et al. (2018) to accurately predict their responses to heightened future thermal variability, compared to *Trichodesmium* populations in more P-limited areas.

*Trichodesmium* in the oligotrophic ocean is also often limited by iron (Fe), or is co-limited by both P and Fe simultaneously (Berman-Frank et al., 2001; Fu and Bell, 2003; Mills et al., 2004; Walworth et al., 2016). Although effects of Fe limitation on the growth of *Trichodesmium* are beyond the scope of our study, investigations of interactions between this other primary limiting nutrient and climate change variables such as warming are needed to provide better predictions of the physiology and distribution of *Trichodesmium* in the future (Hutchins and Boyd, 2016; Jiang et al., 2018).

## Conclusion

The distinct responses of *Trichodesmium* to thermal variation under two phosphate conditions suggest that the physiological effects of thermal variability on GBRTRL101 strain would depend on ambient nutrient availability. Our results also indicate that the responses of *Trichodesmium* to thermal variability would vary with seasonal (winter/summer) temperature regimes and would be impacted by different intensities (± 2°C/± 4°C) of variation. In addition, this study implies that a thermal performance curve obtained at constant temperatures can be used along with the non-linear averaging model (Bernhardt et al., 2018) to provide accurate predictions of growth rates under fluctuating temperatures when P is replete. However, application of this method under P-limited conditions may require further refinement.

Moreover, the phosphorus use efficiency for nitrogen and carbon fixation was elevated by rising temperature and P limited conditions in this study, suggesting a potential for this strain to maintain fitness despite future warmer, more nutrient-limited conditions. In conclusion, the overall effects of ambient temperature, intensity of thermal variation, nutrient supplies and their interactions should be considered together to accurately predict how the growth and nitrogen fixation of *Trichodesmium* may be altered in the future changing ocean.

## Data Availability

Publicly available datasets were analyzed in this study. This data can be found here: https://www.bco-dmo.org/dataset/756942, https://www.bco-dmo.org/dataset/722913, https://www.bco-dmo.org/dataset/722927.

## Author Contributions

PQ carried out the main experiments and wrote the manuscript. DH and F-XF helped to design the experiments. MH and XW assisted in conducting the experiments. JK helped to process the data of thermal performance curves. DH and PQ were involved in the manuscript writing and editing.

## Conflict of Interest Statement

The authors declare that the research was conducted in the absence of any commercial or financial relationships that could be construed as a potential conflict of interest.
