# Supplementary material for: Distinct Responses of the Nitrogen-Fixing Marine Cyanobacterium Trichodesmium to a Thermally Variable Environment as a Function of Phosphorus Availability
Source: Front Microbiol. 2019 Jun 11;10:1282. doi: 10.3389/fmicb.2019.01282 (PMC6579863; doi:10.3389/fmicb.2019.01282)
Supplement: Supplementary file 1 [file Table_1.DOCX]

Supplementary Material

Table S1. Experimental temperature and phosphate availability treatments. LTP: low temperature phase; HTP: high temperature phase. Constant temperatures of 22°C and 30°C were used, along with variable treatments with these same two mean temperatures. For the mean temperature of 22°C, the “intense” variable treatment consisted of 18-26°C fluctuations, and the “mild” variable treatment was 20-24°C. For 30°C, only one variable treatment (28-32°C) was used. All the thermal treatments were run under P-replete (10 μmol/L) and P-limited (0.2 μmol/L) conditions.

| Temperature setting | Phosphate status | LTP  (first 48 hrs) | HTP  (second 48 hrs) |
| --- | --- | --- | --- |
| Constant 22°C | P-limited | 22°C | |
|  | P-replete |  |  |
| “Intensely” variable 22 ± 4°C | P-limited | 18°C | 26°C |
|  | P-replete |  |  |
| “Mildly” variable 22 ± 2°C | P-limited | 20°C | 24°C |
|  | P-replete |  |  |
| Constant 30°C | P-limited | 30°C | |
|  | P-replete |  |  |
| “Mildly” variable 30 ± 2°C | P-limited | 28°C | 32°C |
|  | P-replete |  |  |
