# Supplementary material for: Distinct Responses of the Nitrogen-Fixing Marine Cyanobacterium Trichodesmium to a Thermally Variable Environment as a Function of Phosphorus Availability
Source: Front Microbiol. 2019 Jun 11;10:1282. doi: 10.3389/fmicb.2019.01282 (PMC6579863; doi:10.3389/fmicb.2019.01282)
Supplement: Supplementary file 2 [file Table_2.DOCX]

Supplementary Material

Table S2. Elemental stoichiometry during the low temperature phase (LTP) and high temperature phase (HTP) in three variable temperature treatments at two P concentrations (10 and 0.2 μmol/L). The values in the table are the average values of each triplicated treatment ± standard deviation.

|  | P-replete | | | P-limited | | |
| --- | --- | --- | --- | --- | --- | --- |
| *“Winter” treatment* | C: N | C: P | N: P | C: N | C: P | N: P |
| LTP  22 ± 2°C | 7.73 ± 0.42 | 131.81 ± 19.52 | 17.04 ± 2.27 | 7.77 ± 0.38 | 253.33 ± 43.5 | 32.63 ± 5.79 |
| LTP  22 ± 4°C | 7.27 ± 0.36 | 122.71 ± 4.9 | 16.90 ± 0.54 | 7.51 ± 0.27 | 313.39 ± 16.89 | 41.72 ± 0.93 |
| HTP  22 ± 2°C | 7.07 ± 0.19 | 120.38 ± 4.38 | 17.03 ± 0.50 | 8.41 ± 0.59 | 430.83 ± 91.62 | 51.61 ± 12.75 |
| HTP  22 ± 4°C | 7.09 ± 0.57 | 146.19 ± 22.22 | 20.55 ± 1.45 | 7.81 ± 0.42 | 370.78 ± 28.45 | 47.45 ± 2.30 |
| *“Summer” treatment* | C: N | C: P | N: P | C: N | C: P | N: P |
| LTP  30 ± 2°C | 5.88 ± 0.14 | 107.71 ± 16.60 | 18.31 ± 2.78 | 6.32 ± 0.55 | 248.66 ± 14.18 | 39.40 ± 1.45 |
| HTP  30 ± 2°C | 6.72 ± 0.46 | 124.18 ± 7.92 | 18.57 ± 2.36 | 7.87 ± 0.71 | 399.86 ± 26.9 | 50.89 ± 1.52 |
